# Competitive exclusion approach using an *E. coli* live vaccine to protect broilers from colonization with ESBL-/ pAmpC- *E. coli*

**DOI:** 10.1038/s41598-025-10279-5

**Published:** 2025-07-08

**Authors:** Diana Vargas, Roswitha Merle, Anika Friese, Uwe Roesler, Caroline Robé

**Affiliations:** 1 Veterinary Centre for Resistance Research, Institute of Animal Hygiene and Environmental Health, Freie Universität Berlin, Berlin, Germany; 2 Veterinary Centre for Resistance Research, Institute of Veterinary Epidemiology and Biostatistics, Freie Universität Berlin, Berlin, Germany

**Keywords:** Antimicrobial resistance, Broiler chicken, *Escherichia coli*, ESBL, pAmpC, Competitive exclusion, Biotechnology, Microbiology

## Abstract

Antimicrobial-resistant bacteria originating from broilers pose an ongoing challenge as they can spread in the environment and food chain. One approach to lower colonization is to administer live bacteria to broilers. A live *Escherichia coli (E. coli)* vaccine, consisting of a single *E. coli* strain used as a competitive exclusion (CE) approach, was evaluated to decrease the colonization with ESBL-/pAmpC- producing *E. coli*. 168 ESBL-/pAmpC- negative Ranger Gold broilers were divided into six groups (3 x *n* = 46 experimental groups; 3 x *n* = 10 control groups for quality assurance). The *E. coli* vaccine was administered on day one through coarse spray or by drinking water on day five. Experimental groups were orally co-colonized with 10^2^ cfu of one ESBL- (ST410, *bla*_CTX−M−15_) and one pAmpC- producing *E. coli* (ST10, *bla*_CMY−2_/*mcr*-1) on day three. Colonization status was monitored throughout the trial and quantified at the end of the study (day 49). A transient reduction in ESBL-producing *E. coli* colonization (*p* < 0.001) was observed following coarse spray administration. However, this decrease was not sustained over time. It can be concluded that a single *E. coli* strain originating from a live vaccine cannot decrease colonization of broilers with ESBL-/pAmpC- *E. coli* throughout a fattening period.

**Supplementary Information:**

The online version contains supplementary material available at 10.1038/s41598-025-10279-5.

## Introduction

Antimicrobial resistant bacteria originating from livestock represent an ongoing challenge due to their potential spread to the environment and the food chain, ultimately posing risks to human health through environmental contamination or foodborne transmission^1,2^. Moreover, poultry production has been recognized as a major reservoir for Extended-Spectrum Beta-Lactamase- (ESBL-) and plasmid-mediated AmpC Beta-Lactamase- (pAmpC-) producing *Enterobacterales*^3,4^. ESBL- and pAmpC enzymes confer resistance to a large variety of Beta-lactam antibiotics, including penicillins, third-generation cephalosporins, and monobactams^5^. Resistant bacteria have been found throughout the broiler production chain, from early breeding stages to retail products, highlighting their widespread presence and potential transmission^6–9^.

The poultry production chain is highly interconnected, allowing unwanted bacteria to enter at various stages^8,10^. Different interventions and improvements at each level have been investigated to reduce or prevent colonization. Farm practices, including hygiene barriers, as well as regular cleaning and disinfection, have demonstrated potential in the reduction of bacterial loads^11,12^. Additionally, on-site management strategies, such as implementing alternative housing systems, selecting different breeds, and adjusting stocking densities have also been investigated to mitigate the problem^13^. Even though some of these approaches have been shown to lower the number of undesirable bacteria, they still do not fully suppress them^9,11,14^. The effect of probiotic treatments based on a competitive exclusion (CE) approach has also been employed, showing significant effects at reducing the colonization by ESBL-/pAmpC- producing *Escherichia coli (E. coli)* in broiler flocks^15,16^.

In poultry farming, CE refers to the process by which beneficial bacteria are administered to the newly hatched birds to colonize their gut, inhibiting the colonization of harmful, pathogenic microorganisms^17^. These products are typically derived from complex intestinal communities of adult animals, making full characterization difficult^18^. Although the exact mechanisms by which these cultures work remains unclear due to the gut complexity, proposed actions include receptor competition, direct or indirect competition for nutrients, production of antimicrobial compounds, and host’s immune system stimulation^19^. Even though CE products have been shown to reduce colonization by pathogenic or antimicrobial-resistant bacteria in broiler chickens such as *Salmonella spp.* or *E. coli*^15,20^, their use remains limited in some countries, including Germany^18,21^, due to concerns over their undefined or unstandardized bacterial composition^21,22^. Based on this limitation, we hypothesized that a live *E. coli* vaccine used with a CE approach could reduce or prevent colonization by ESBL- and pAmpC- producing *E. coli* in broiler chickens.

## Results

### ESBL-/pAmpC- *E. coli* colonization during trial

**Fig. 1:**
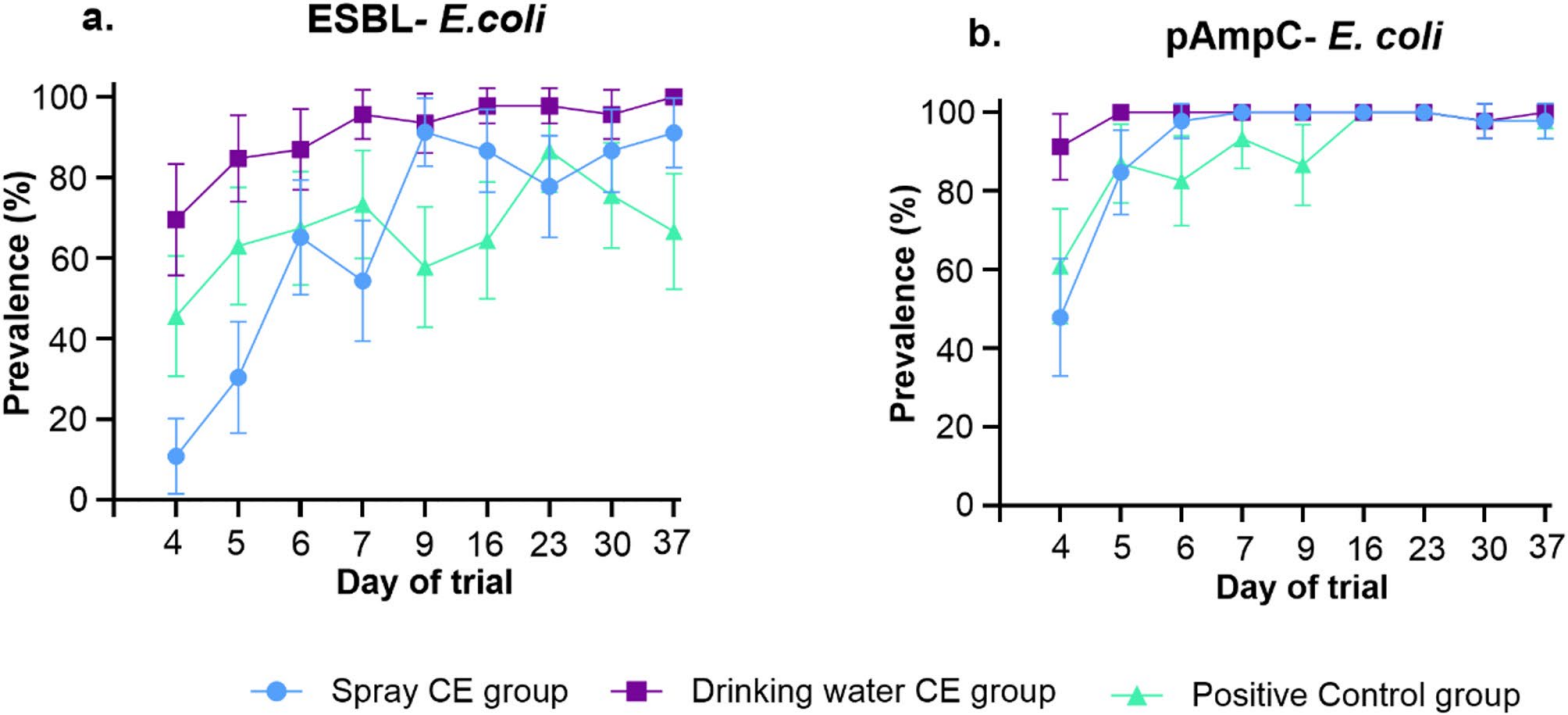
(**a**) ESBL- and (**b**) pAmpC- producing *Escherichia coli (E. coli)* prevalence throughout the trial in the groups administered the competitive exclusion (CE) by coarse spray, via the drinking water, and positive control group. Error bars display 95% confidence intervals. Groups were orally co-inoculated with ESBL- and pAmpC- producing *E. coli* on day three of life.

The colonization of ESBL-and pAmpC- producing *E. coli* strains in each broiler was tracked throughout the entire study by collecting cloacal swabs, beginning on the first day of life. Following co-inoculation on day three of life, colonization prevalence varied by strain, indicating a strain-specific colonization pattern (Fig. 1, S. Fig. 1).

A gradual increase in the prevalence of the ESBL- producing *E. coli* strain was observed. Broilers receiving the CE via drinking water on day five reached over 70% positivity by the first day post-inoculation, and more than 95% by day seven of life (fourth day post-inoculation). In contrast, CE administered via coarse spray on day one delayed colonization, resulting in significantly lower prevalence on days one and two post-inoculation (mixed logistic regression model *p* < 0.001), though differences were no longer significant thereafter. Broilers from this group reached a prevalence of 95% by day nine (fifth day post-inoculation). The positive control group showed the lowest increase in prevalence for the ESBL- producing *E. coli* strain, with over 80% of the broilers testing positive by day twenty-three of life (twentieth day post-inoculation) (Fig. 1a). The pAmpC- producing *E. coli* strain showed a rapid increase in prevalence, with approximately 80% of broilers across all groups testing positive by day five (second day post-inoculation) (Fig. 1b.). A lower prevalence could be observed in the experimental group administered the CE via coarse spray when compared to the positive control group on the first day post-inoculation, however, it is not statistically different (mixed logistic regression model *p* = 0.065). Neither ESBL- nor pAmpC- producing *E. coli* were detected in broilers prior to inoculation on day three. All non-inoculated control groups remained negative throughout the study.

### ESBL-/pAmpC- *E. coli* quantification at necropsy

After a fattening period of 49 days, the content of the cecum and colon of every broiler was collected and analyzed respectively. The administration of the CE, on day one by coarse spray or on day five through the drinking water, did not reduce the bacterial loads of both ESBL-/pAmpC- *E. coli* strains in either the cecum or the colon samples compared to the positive control group.

**Fig. 2:**
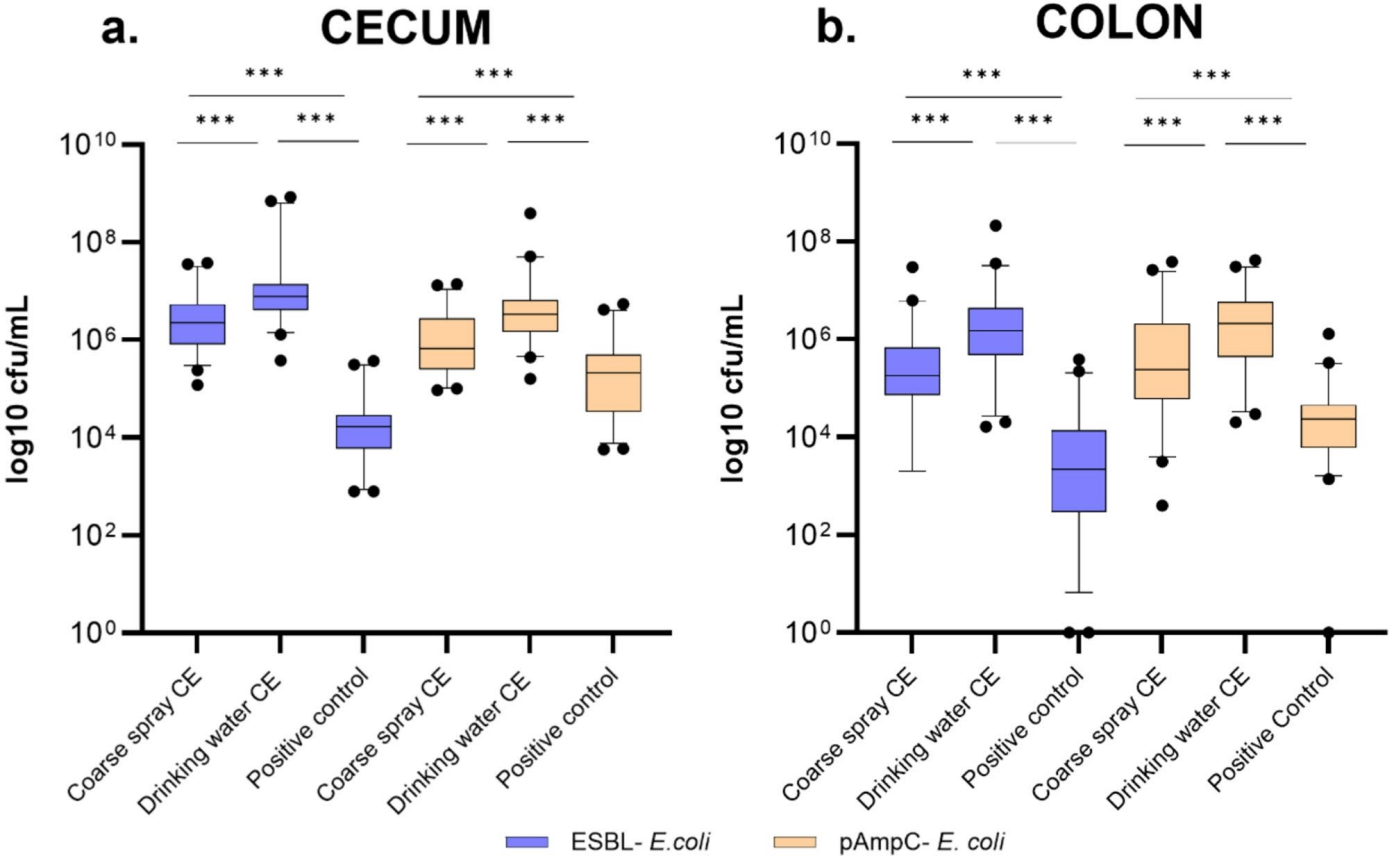
Colonization of broiler chickens with ESBL- and pAmpC- producing *Escherichia coli (E. coli)* in (**a**) Cecum and (**b**) Colon in 3 investigated groups: group administered the competitive exclusion (CE) by coarse spray, group administered the CE via the drinking water, and positive control group. Colonization levels were determined at necropsy. *** *p* < 0.001 (ANOVA, Tukey’s (HSD). Boxes represent the interquartile range, with a horizontal line inside indicating the median. Whiskers extend from the 5th to the 95th percentile, excluding outliers, which are shown as individual points.

In both the cecum and colon, broilers receiving the CE product showed significantly higher bacterial counts of ESBL- and pAmpC- producing *E. coli* compared to the positive control group (Tukey’s Honest Significant Difference (HSD) *p* < 0.001). Among the experimental groups, CE administration via drinking water on day five resulted in significantly greater colonization than coarse spray application on day one (*p* < 0.001) (Fig. 2).

Cecum and colon samples from the control groups exposed to the CE either through coarse spray on day one or drinking water on day five, and not co-inoculated with the ESBL-/pAmpC- producing *E. coli* strains, along with the negative control group, tested negative.

## Discussion

We investigated the effect of a live, attenuated *E. coli* vaccine strain on its ability to prevent or decrease the colonization of ESBL- and pAmpC- producing *E. coli* in broiler chickens using a CE approach. Our findings show that coarse spray administration on the first day of life leads to a short-term reduction in ESBL- producing *E. coli* for up to two days after inoculation, but this effect is not maintained over time. Additionally, no reduction was observed for the pAmpC- *E. coli*. Similarly, administering the single *E. coli* strain through drinking water on day five did not lead to any decrease in the targeted bacteria.

This study employed a single, defined *E. coli* strain within a CE approach using approved coarse spray and drinking water application methods. Single-culture products are favored for their regulatory compliance, standardized composition, and reproducibility^18,21^, whereas multi-strain formulations, typically derived from complex intestinal cultures, face regulatory restrictions in countries like Germany due to their inconsistent composition^21,22^. Their complexity makes definition and characterization challenging, thereby limiting their regulatory approval^18,23^. Although classified by the WHO as “normal gut flora” rather than vaccines or veterinary drugs, CE products in the EU must meet strict characterization standards under Regulation (EC) No 1831/2003^22^. Despite regulatory challenges, multi-culture CE formulations have demonstrated superior efficacy^23^, likely due to broader microbial diversity enhancing competitive pressure against pathogens^22,24^. In this study, however, the use of a single *E. coli* may not have been sufficient to successfully inhibit the colonization of the inoculated antibiotic-resistant strains, possibly needing the presence of additional bacterial species to better inhibit the growth of unwanted bacteria and reduce the occupation of available niches.

In our trial, the *E. coli* strain with a CE approach was given to the chickens following the manufacturer’s recommendations for broilers, involving a single application either via coarse spray on day one or through the drinking water on day five. However, no sustained reduction in colonization of ESBL- or pAmpC- producing *E. coli* was observed. This lack of long-term effect may be partly due to the absence of repeated dosing. For example, Wang, et al.^25^ reported a reduction in *E. coli* K88 counts in the cecal contents of broiler chickens following continuous dietary supplementation with *Lactobacillus plantarum* B1 over a 28-day trial period. This suggests that repeated application of CE cultures may be necessary to stablish and maintain protective colonization. Furthermore, reapplication could counteract shifts in the microbiota caused by dietary or environmental changes^26^, which can destabilize the microbial balance and diminish the persistence of beneficial strains. Therefore, the lack of protection in our study may reflect the limitations of a single-dose strategy.

The analysis of the cloacal swabs showed an initial two-day reduction in the prevalence of the ESBL- producing *E. coli* strain in the group administered the CE by coarse spray on day one of life, following a prophylactic approach. These results align with findings from Methner, et al.^27^, who used a commercial *S. typhimurium* vaccine with a CE approach to prevent *S. typhimurium* colonization. In the trial, the vaccine was given orally on hatch day and challenged in the same way the next day. While a decrease in the inoculation of *S. typhimurium* is noted, the exclusion effect was only evident initially post-inoculation, after which the levels of the unwanted *S. typhimurium* increased. This indicates that when live attenuated vaccines are used with a CE with a prophylactic approach, an initial protection can be observed.

No reduction or inhibition was observed for any of the ESBL-/ pAmpC- producing *E. coli* inoculation strains in the group administered the CE strain by drinking water on day five of life. This experimental group was given the product with an intervention approach, two days after the chicken were orally co-inoculated. This suggests that post-challenge administration was too late to be effective, highlighting the limitations of a treatment-based approach. According to Ballou, et al.^28^ and Diaz Carrasco, et al.^29^, the initial development of the gut’s microbiome in poultry is crucial for overall health and productivity. The development of the microbiome is a dynamic process that begins at hatching and evolves throughout the chicken’s life, being highly influenced by certain factors such as the environment, diet, and management practices^26^. Based on this, it is recommended that any microbiome modulation intended to be done gives its best effects when it is done at the earliest possible time, when it is still in an immature state, and prior to any challenge^22,30^. Considering also that compared to natural intestinal bacteria, unwanted bacteria tend to colonize and establish more rapidly, perhaps outcompeting beneficial bacteria^31^. As pointed out in studies by Stanley^32^, some variability can also be observed in the gut’s microbiome among the birds, even under the same controlled conditions. Despite our efforts to handle and keep all the animals under identical settings throughout the trial, some variations may have arisen due to the inter-individuality among the birds.

## Conclusion

In conclusion, even though CE cultures have shown to be effective, the regulation around their use is limiting the options for the poultry industry in some countries. Our results show that the use of an already approved live *E. coli* vaccine, when applied with a CE approach, does not decrease or prevent the colonization of ESBL-/pAmpC- producing *E. coli* in broilers. However, when administered on the first day of life via coarse spray with a prophylactic CE approach, an initial decrease in the colonization of ESBL- producing *E. coli* in broilers is observed. This effect may be further explored to enhance early colonization resistance and prevent displacement by undesirable bacteria. Furthermore, this approach provides a basis for evaluating other defined, approved, and well-characterized products or individual strains.

## Methods

### Ethics statement

This study was conducted in compliance with the National Animal Protection Guidelines. The experiment protocol received approval from the German Animal Ethics Committee for the protection of animals of the Regional Office for Health and Social Affairs Berlin (“Landesamt für Gesundheit und Soziales”, LAGeSo, registration G0079/23). The trial adhered to the national and organizational regulations of Freie Universität Berlin concerning the care and use of animals. The experimental protocols, aimed at causing only minor discomfort to the animals, received approval from LAGeSo. The study complied with ARRIVE guidelines.

### Broiler chickens

The trial was carried out at the experimental animal facility of the Centre for Infection Medicine of Freie Universität Berlin, within the School of Veterinary Medicine. Disinfected eggs from the slower-growing Ranger Gold broiler breed, which has an average weight gain of 45 g per day, were obtained from a commercial hatchery in Germany. Upon arrival at the facility, all eggs were disinfected using WESSOCLEAN^®^ Gold line (Wesso AG, Hersbruck, Germany). For the first 18 days, eggs were incubated at 37.8˚C and 60% relative humidity. Between days 18 and 21, the temperature was reduced to 37.2˚C, and the humidity increased to 80% to support the hatching process. The sample size for the animal trial was determined based on a power analysis. An analysis of variance (ANOVA) with multiple post hoc comparisons was planned, targeting 80% statistical power and a 95% confidence level. The calculation assumed a minimum difference of 1 log between the control group and each of the two experimental groups, with an estimated standard deviation of 1 log. Immediately after hatching, chickens were randomly allocated into six groups and individually tagged (Table 1).

### Housing conditions and management

Prior initiation of the trial, all rooms and equipment were cleaned and disinfected with hydrogen peroxide and tested for the absence of ESBL-/pAmpC- producing enterobacteria. Sterile gauze swabs moistened with 5 ml of Phosphate-Buffered Saline (PBS) (Oxoid, Wesel, Germany) were employed to sample multiple surfaces, including feeders and drinkers. Additionally, samples of litter and feed were collected. All samples were transferred into 50 ml of Luria-Bertani broth (LB) (Carl Roth, Karlsruhe, Germany) and incubated at 37 °C for 24 h. Thereafter, 10 µl were streaked onto chromogenic agar plates supplemented with 2 µg/ml cefotaxime (CHROMagar Orientation, Mast Diagnostica, Reinfel, Germany) and incubated for 24 h at 37 °C to screen for the presence of ESBL-/pAmpC- producing bacteria.

Animals were housed in separate experimental units, equipped with individual HEPA filters for ventilation and a designated anteroom for changing clothes. Except for the interventions being tested, all groups were kept and treated under identical conditions. Housing conditions included a stocking density of 21 kg/m², fresh litter (1 kg/m²) provided once at the beginning of the trial, pecking stone blocks, and spelt husk briquettes. A light schedule was established with alternating light and darkness periods and included a dimming phase. The room temperature gradually reduced, starting at 28˚C from days 1 to 8, then to 26˚C from days 8 to 16, followed by 24˚C from days 17 to 24, and finally reaching 22˚C until the end of the trial, with a maintained relative humidity of 55%. Broilers were given a starter feed for the first two weeks, a grower feed until day 40, and a finisher feed for the final 9 days, with water provided *ad libitum*.

### Administration of competitive exclusion: live *E. coli* strain

Poulvac *E. coli* (Poulvac^®^, Zoetis, USA) is an attenuated live *E. coli* vaccine, commercially available for broilers, layers, breeders, and turkeys as a preventative measure against colibacillosis. According to manufacturer, the vaccine is administered in the field using one of the two approved methods: either by coarse spray at day one or by drinking water at 5 days of age. Both approved applications were tested in the trial with a CE approach, based on the idea that the vaccine’s live attenuated *E. coli* strain, when provided in early stages of life, will reduce or give protection against colonization of the antimicrobial resistant bacteria.

The vaccine was applied following the manufacturer’s instructions. The coarse spray experimental group and coarse spray control group received the vaccine on the day of hatch by coarse spray using a hand sprayer pump (Gloria Hobby 10 FLEX, Witten, Germany). The coarse spray application was performed two days prior to co-inoculation with ESBL-/pAmpC- producing *E. coli* strains and thus was evaluated as a preventive measure (Table 1). The drinking water experimental group and drinking water control group received the vaccine via drinking water on the fifth day of life, evaluated as an intervention strategy following oral co-inoculation with the ESBL-/pAmpC- producing *E. coli* strains (Table 1). To ensure an adequate consumption of the vaccine, consisting of a single live *E. coli* strain, birds were restricted from accessing water for two hours prior to water vaccination. The positive control group and negative control group did not receive the vaccine.

### Bacterial strains and oral co-inoculation

For ESBL-/pAmpC- producing *E. coli* colonization of the broiler chickens, two *E. coli* strains isolated from healthy chickens during a former research project were used, with whole-genome sequencing employed to characterize their genetic profiles^33,34^. An ESBL- producing *E. coli* strain, which carries the *bla*_CTX−M−15_ gene chromosomally encoded^35^, ST410, phylogenetic group A, resistant to cephalosporins and enrofloxacin^33^, and a pAmpC-*E. coli*, ST10, phylogenetic group A, *bla*_CMY−2_ plasmid encoded^35^, resistant to cephalosporins and colistin. The bacterial suspension used to inoculate the animals was prepared following the method described by Robé, et al.^36^. Briefly, both bacterial strains were streaked out on Columbia agar with 5% sheep blood (Oxoid, Wesel, Germany) and incubated overnight at 37 °C. Single colonies were then used to inoculate 3 mL of LB broth, which was incubated overnight at 37 °C under shaking conditions. Next day, 60µL of each bacterial suspension was transferred into 4 mL of fresh LB broth and left in shaking incubation at 37 °C until the cultures reached an optical density at 600 nm (OD_600_) of 1.0. Cultures were subsequently washed with PBS, and the desired final concentrations were prepared through serial dilutions. For co-inoculation of the birds, the prepared bacterial suspensions were thoroughly mixed and kept on ice until use, ensuring they were utilized within 30 min. The bacterial count in the suspension was verified by appropriate plating.

On day three, all animals within the experimental groups, including the positive control group, were orally co-inoculated with an equal mixture of both bacterial strains at a concentration of 10^2^ cfu (Table 1). A very low dose of colonization was employed, as previous evidence indicates that it is sufficient to achieve colonization in the birds^36^. Each bird received a total of 200µL of the bacterial mix suspension via a crop needle. The control groups, including the negative control group, received 200µL of PBS to ensure a comparable treatment across all birds within the groups.

### Sampling design

During the first three days of life, cloacal samples were taken from all animals to assure ESBL-/pAmpC- producing *E. coli* absence. Swabs (SARSTEDT AG & CO. KG, Germany) were each transferred into tubes with 500µL PBS, thoroughly homogenized, and 50µL were plated on chromogenic agar without antibiotic supplementation for the detection of any *E. coli* colonies and on chromogenic agar with 2 µg/ml cefotaxime to confirm absence of ESBL-/pAmpC- producing bacteria before co-inoculation of the broiler chickens on day three. To verify negative results of ESBL-/pAmpC- producing bacteria, samples were further enriched in LB, incubated overnight at 37^◦^C, and re-plated on chromogenic agar with 2 µg/ml cefotaxime.

Cloacal swabs were collected to monitor the colonization status of broiler chickens with ESBL-/pAmpC- producing *E. coli.* Sampling began on day four of life and continued daily until day eight, followed by weekly sampling on days 9, 16, 23, 30, and 37 of life. Swabs were processed using the procedure detailed above and plated on one plate supplemented with 2 µg/ml cefotaxime as a control for the overall ESBL-/pAmpC- producing *E. coli*, a plate containing 2 µg/ml cefotaxime and 4 µg/ml enrofloxacin for detection of the ESBL- producing *E. coli* strain. A plate with 2 µg/ml cefotaxime and 7 µg/ml colistin for growth of the pAmpC- producing *E. coli* strain, and a plate with all three antibiotics in the given concentrations that served as negative control. Plates were incubated for 24 h at 37^◦^C for semi-quantitative analysis (Categories 0 to 4. 0 = no growth, 1 = ≤ 10 cfu *E. coli*, 2 = 11 to 100 cfu *E. coli*, 3 = 101 to 200 cfu *E. coli*, 4 = ≥ 201 cfu *E. coli* (S. Fig.  1)). In instances where atypical morphology of *E. coli* colonies was observed, MALDI-TOF/TOF (UltrafleXtreme^®^; Bruker Daltonics GmbH & Co. KG, Bremen, Germany) was used to provide further confirmation.

After a fattening period of 49 days and a targeted weight of 2.2 kg, all animals underwent necropsy. For sedation, a combination of ketamine hydrochloride (Ketabel 100 mg/ml 25 ml, BelaPharm GmbH & Co. KG., Germany), xylazine hydrochloride (Xylazin 20 mg/ml (Xylazin 2%) 25 ml Serumwerk Bernburg), and midazolam hydrochloride (MIDAZOLAM-ratiopharm 15 mg/3 ml Ratiopharm GmbH, Germany) was injected into the broiler’s chest muscle. After confirmation of deep sedation, broilers were sacrificed by cervical dislocation. For ESBL-/pAmpC- producing *E. coli* quantification, cecum and colon content was collected in separate reaction tubes per each broiler chicken and PBS was added to a ratio 1:2. Dilution series in PBS were performed, and 50µL were plated on the plate set described before and incubated for 24 h at 37^◦^C for quantitative analysis.

### Statistical analyses

All statistical analyses were conducted using IBM SPSS Statistics version 25.0 for Windows (SPSS, Inc., Chicago, IL, USA). Colonization by each strain was analyzed using a mixed logistic regression model, with “individual animal” as the observational unit and “experimental group” as a random effect.

To compare colonization levels in the cecum and colon between the different groups, an analysis of variance was conducted, including Tukey’s Honest Significant Difference (HSD) test for multiple comparisons. The level of significance was set at *p* < 0.05. Assumptions of normality and homogeneity of variances of residuals were checked after performing the test.

**Table 1:** Experimental design: Animal groups, ways of and time points for competitive exclusion (CE) administration, and co-inoculation with ESBL-/pAmpC- producing *Escherichia coli (E. coli)*.

Day of trial	Coarse spray CE group	Drinking water CE group	Positive control group	Coarse spray CE control group	Drinking water CE control group	Negative control group	Cloacal swab sampling
	46 animals	46 animals	46 animals	10 animals	10 animals	10 animals	
1	CE administration by coarse spray	–	–	CE administration by coarse spray	–	–	ESBL-/pAmpC- producing *E. coli* absence
3	Oral co-inoculation with ESBL-/pAmpC- producing *E. coli*			–	–	–	
5	–	CE administration by drinking water	–	–	CE administration by drinking water	–	Colonization status of ESBL-/pAmpC- producing *E. coli*
6–48*							
49	Necropsy: Quantification of ESBL-/pAmpC- producing *E. coli* in cecum and colon						

* Weekly cloacal sampling on days 9, 16, 23, 30, and 37 of life.

## Electronic supplementary material

Below is the link to the electronic supplementary material.

Supplementary Material 1

## Data Availability

Semi-quantitative measurement of the colonization of ESBL-/ pAmpC- producing *E. coli *determined by cloacal swabs throughout the trial is provided in Supplementary information Figure 1. Data generated and/or analyzed during this study are available upon reasonable request from Vargas, D. or Friese, A.
