# Supplementary material for: Competitive exclusion approach using an E. coli live vaccine to protect broilers from colonization with ESBL-/ pAmpC- E. coli
Source: Sci Rep. 2025 Jul 8;15:24547. doi: 10.1038/s41598-025-10279-5 (PMC12238256; doi:10.1038/s41598-025-10279-5)
Supplement: Supplementary file 1 — Supplementary Material 1 [file 41598_2025_10279_MOESM1_ESM.pdf]

Supplementary information

Competitive exclusion approach using an *E. coli* live vaccine to protect broilers from colonization with ESBL-/ pAmpC *E. coli*

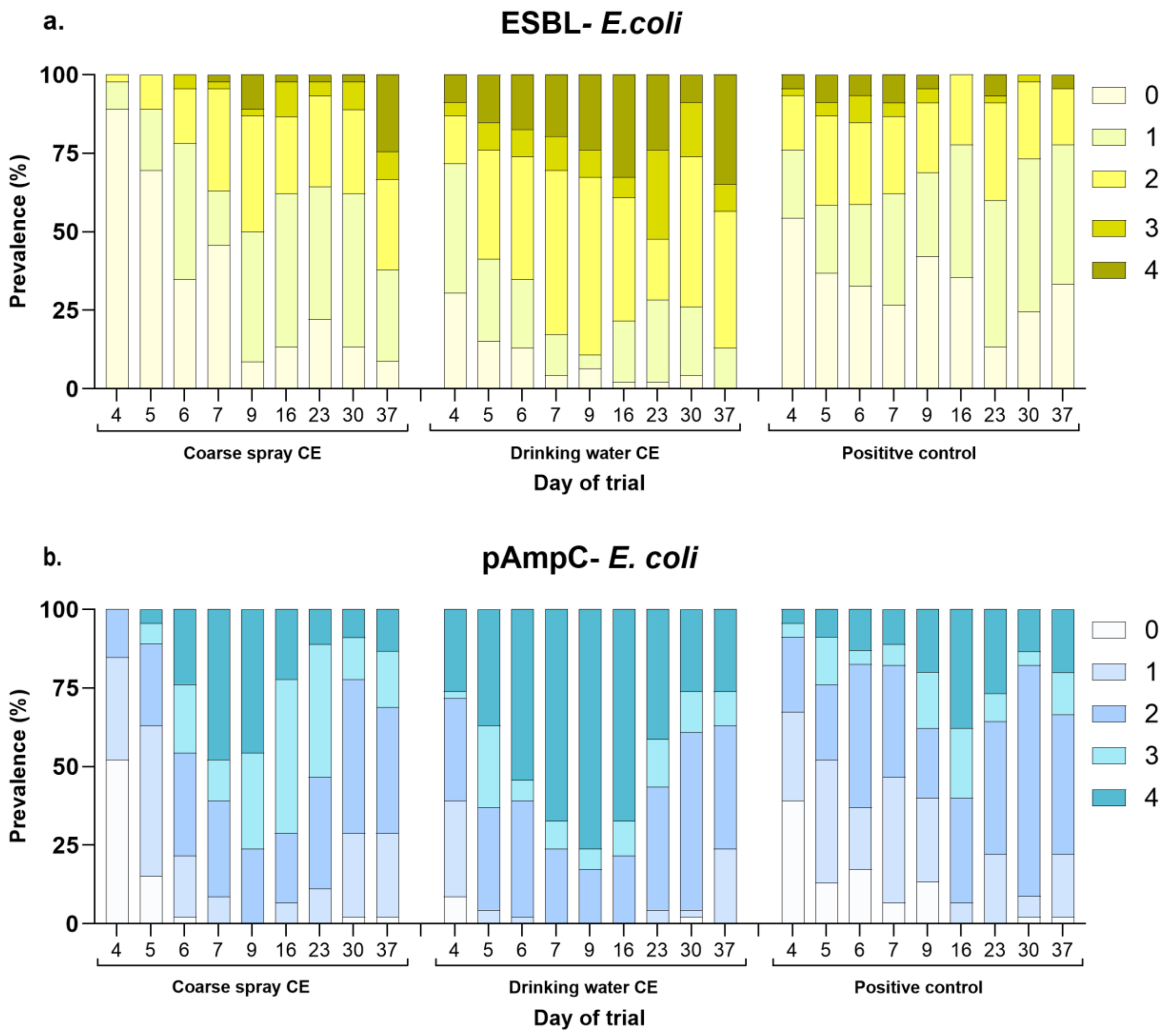

S. Figure 1: Semi-quantitative measurement (categories 0 to 4). 0 = no growth, 1 = ≤ 10 cfu *E. coli*, 2 = 11 to 100 cfu *E. coli*, 3 = 101 to 200 cfu *E. coli*, 4 = ≥ 201 cfu *E. coli* of the colonization of a. ESBL-producing *E. coli* and b. pAmpC- producing *E. coli* determined by cloacal swabs throughout the trial.
